# Factors influencing utilization of modern family planning services by persons living with Human Immunodeficiency Virus at Luwero Hospital, Uganda

**DOI:** 10.4314/ahs.v22i3.50

**Published:** 2022-09

**Authors:** Kizito Omona, Geoffrey Muhanuzi

**Affiliations:** 1 Lecturer, Uganda Martyrs University, Faculty of Health Sciences, Kampala, Uganda; 2 MPH Specialist, Kampala University, Department of Public Health, Kampala, Uganda

**Keywords:** Mother-to-Child Transmission (MTCT), HIV, Family Planning, Unintended Pregnancy, Luwero district

## Abstract

**Introduction:**

The use of modern family planning methods is key for achieving the prevention of unintended pregnancies among women living with HIV, in the prevention of Mother-to-child transmission (PMTCT) package. The purpose of this study was to examine the factors influencing the utilization of modern family planning services by persons living with HIV at Luwero Hospital, Uganda.

**Methods:**

The study was conducted among 210 persons living with HIV attending the ART clinic and was based on cross-sectional descriptive and analytical design. Sampling was by simple random techniques. Data was collected using researcher-administered questionnaires.

**Results:**

The uptake of Modern FP services is low (36.7%) among persons living with HIV. It was attributed to client-related factors such as being married [AOR: 2.2, 95% CI [1.123–4.140], p = 0.038]) and other factors. These are; religious views discouraging use of modern FP (p= 0.034), negative side effects (AOR: 1.8, 95% CI [0.043–1.968], p = 0.044) and services being unfriendly for persons living with HIV (p=0.000]).

**Conclusions:**

Despite the presence of modern family planning services, uptake among persons living with HIV is low. Poor utilization is a recipe for unintended pregnancy and thus jeopardizes efforts in the elimination of mother-to-child transmission of HIV.

## Introduction

### Background of the Study

According to the World Health Organization (WHO), HIV infection is one of the biggest public health challenges the world has ever seen in recent history and it continues to be a major public health problem globally^1^. The same WHO report indicates that many of the affected individuals are in sub-Saharan Africa where 25.6 million people live with Human Immunodeficiency Virus (HIV). However, the United Nations Organization on acquired immunodeficiency syndrome [AIDS] (UNAIDS) reveals that while there has been commendable reduction in new HIV infections by about 41% between 2000 and 2015, women of reproductive age still account for about two-thirds of new HIV infections globally and in sub-Saharan Africa 2. The high rate of new HIV infections among women of reproductive age presents stumbling block in the prevention of mother-to-child transmission (PMTCT) of HIV 1, 2. The use of modern family planning methods is key for achieving the prevention of unintended pregnancies among women living with HIV (prong 2), in the prevention of Mother-to-child transmission (PMTCT) package. This prong emphasizes the prevention of unwanted pregnancies among women living with HIV, which tremendously contribute to the PMTCT of HIV infection 3.

Further, the use of contraceptives among HIV-infected women (prong 2) has the potential of preventing over 173,000 unintended HIV-infected births each year in Sub-Saharan Africa^4^. In addition, the use of contraceptive services has numerous other benefits among HIV-infected women, such as leading to the reduction in morbidity and mortality rates due to pregnancy, and also leading to improvements in the health of HIV-infected women by reducing unintended pregnancies^5^. Similarly, the use of contraceptives, mainly the condoms, provides dual protection as it protects against unintended pregnancy and acquisition or transmission of other sexually transmitted infections, including other strains of HIV^5^. However, while more than 90% of HIV cases occur in Sub-Saharan Africa^6^, the use of modern family planning services is still lowest among women living with HIV^7^.

Globally, modern contraceptive utilization has increased in the recent past – from 54% in 1990 to 57% in 20128. However, the estimates in Africa remain persistently low at 23% in 1990 and 24% in 2012, respectively^8^. The estimates among countries in the Sub-Saharan region are much lower than the aforementioned figures. In 2019, there were about 1.9 billion women of child-bearing age (15–49 years) worldwide. Globally, 1.1 billion need family planning; of these, 842 million are currently using contraceptives, while 270 million have an unmet need for contraception. The current global estimate for the need for family planning satisfied by modern methods based on Sustainable Development Goals (SDG) indicator 3.7.1 was 75.7% in 2019; yet less than half of the need for family planning was met in Middle and Western Africa6. Among persons living with HIV (PLHIV), the utilization of family planning services is highest in the developed world; in countries like the United States of America, the United Kingdom, Japan, China, Canada, and other developed countries around the globe. These countries also have the lowest rates of mother-to-child transmission of HIV^6^.

Sub-Saharan Africa has the highest global prevalence of HIV and yet it also has the highest prevalence of unmet need for modern family planning services^9^. Further study, according to Ogbe and Mutihir^10^ on the use of family planning services, shows a much lower result among PLHIV than their non-HIV infected counterparts. In South Africa, a cohort study by Schwartz et al^11^ indicated that nearly one in four women had at least one unplanned pregnancy within two years of initiating ante-retro viral therapy (ART) and that 62% of the pregnancies were unplanned. In Ethiopia, Ferid et al^12^ reported that the unmet need for modern family planning services is very high among HIV-Positive Women. In Uganda, there have been commendable efforts aimed at improving the access to and uptake of modern family planning services. Over the years, the government of Uganda has committed to scale up the use of modern family planning methods to ensure that every Ugandan woman can choose when and how many children to have. In 2017 it revised its original commitment of 2012 to reduce the unmet need among adolescents from 30.4% in 2016 to 25% in 2021. By improving the number of health structures in hard-to-reach places, the Government of Uganda strives to expand its reach and provision of services and method mix, including long acting, reversible, and permanent methods. These commitments have resulted in several gains, with the Uganda Demographic and Health Survey (UDHS) reports indicating an increase in the uptake of modern contraceptive methods among married women from 14% in 2000/2001 to 35% in 2016, although this report wasn't disaggregated by HIV status^13^. Nonetheless, research report by Alhassan et al^14^ indicated that in Uganda, the unmet need for family planning services is very high among PLHIW. Further, an analytical study by Omona and Namuli^15^ about the factors influencing utilization of intra-uterine device among postpartum mothers in the nearby Butambala district, proved that the uptake of contraceptive services is low to the point that the prevalence of intrauterine contraceptive method was low at 16.3%. The broad objective of this current study was to examined the factors influencing the utilization of modern family planning services by persons living with HIV (PLHIV) at Luwero Hospital in Luwero District.

### Specific Objectives

1. To find out the client-related factors influencing the utilization of modern family planning services by persons living with HIV at Luwero Hospital in Luwero District.

2. To determine the community factors that influence the utilization of modern family planning services by persons living with HIV at Luwero Hospital in Luwero District.

3. To examine method-related factors influencing the utilization of modern family planning services by persons living with HIV at Luwero Hospital in Luwero District.

4. To explore the service provision-related factors that influence the utilization of modern family planning services by persons living with HIV at Luwero Hospital in Luwero District.

## Methods

### Study Design

The study used cross sectional descriptive and analytical designs. Cross-sectional design is characterized by the collection of relevant information (data) at a given point in time. The study employed quantitative since quantitative data was collected in form of nominal, categorical and continuous data.

### Study Area

This study was carried out at Luwero Hospital, located in Luwero Town, Luwero District, Central Uganda, at a distance of about 75 kilometres from Kampala City by road, along Kampala – Gulu Highway. Luweero District is bordered by Nakasongola District to the north, Kayunga District to the east, Mukono District to the southeast, Wakiso District to the south, and Nakaseke District to the west. This hospital is the main referral hospital in the district, and provides preventive, diagnostic, curative, and rehabilitative services. The hospital runs an ART clinic which provides diagnostic and treatment services for PLHIV. According to the 2014 National Population and Housing Census, Luwero District has a population of 458,158 people. This district forms part of Bulemeezi Ssaza, one of the counties of Buganda Government. A report by the Luwero District HIV Focal Person indicate that 23,131individuals were tested for HIV at Luwero Hospital, and 1,565 tested HIV positive (6.8%). This is higher than the Uganda national HIV prevalence of 6.2% 16.

### Study Population

The study population was PLHIV (women and men) of reproductive age, attending the ART clinic at Luwero Hospital.

### Eligibility Criteria

#### Inclusion

The study included PLHIV who were attending ART clinic at Luwero Hospital who were at least 18 years of age since this is the age of consent in Uganda, and who voluntarily accepted and consented in writing to participate in the study.

#### Exclusion

The study excluded those who were not present at the time of the study and those who were not in the right health or psychological mood, and those who had any other challenges that affected their ability to concentrate or offer the information required for the study.

### Sample Size Determination

This sample size was determined using the Kish Leslie formula (1965)17 for cross sectional studies:

n =

Thus, n = 210 respondents

Where;

n = the sample size

### Sampling procedures

A simple random sampling method was used to select the study participants. The daily clinic attendance register was used to prepare the sampling frame comprising of men and women in reproductive age. Pieces of papers of the same size, colour, shape and texture were used according to the number of ‘would be respondents' on the sampling frame. The papers were folded and placed in a small box and shaken. A ‘would-respondent’ was requested to pick a piece of paper without returning. If the picked piece of paper had any of the numbers 1–21 written on it, then the recipient/respondent was considered for the study given that he/she met the inclusion criteria.

### Data Collection

Researcher-administered questionnaire method was used for obtaining data from the primary respondents (PLHIV). This was because most of the study respondents were not comfortable with reading and writing in English language. Therefore, the researcher/research assistant asked the questions in the local language and documented the responses on the data collection tool. The interviews were conducted in private for each respondent in order to foster privacy and confidentiality.

### Data Analysis

Data from the questionnaires was sorted out and coded accordingly, prior to analysis using SPSS version 16.0. Descriptive statistics was used to generate simple descriptive information such as proportion and frequencies. Pearson's Chi-square statistic and multivariate logistic analysis was done to examine the significance of each variable in influencing the utilization of modern family planning services by PLHIV. The significant level for all statistical analyses was set at p≤ 0.05.

### Ethical considerations

The Uganda Martyrs University Research ethics committee and Faculty of Health Sciences reviewed the proposal, the informed consent forms, the letter granting permission to the data collection site, and the interview questions. All the necessary ethical approvals were sought. Prior to seeking informed consent, participants were provided with information about the study, thereafter, participants were given a written informed consent. Participation in the study was voluntary. The information provided by the participants was kept confidential and would not be shared with a third party.

## Results

### Background Characteristics of Individuals

A total of 210 PLHIV attending the ART clinic of Luwero Hospital participated in the study. Basing of the results in table 1 above, most of whom, 115(54.8%) were aged 20 – 29 years, female, 176(83.8%), of secondary level education, 90(42.9%), married, 133(63.3%), of protestant religion, 76(36.2%), and self-employed, 128(61.0%). Further, the study results in table 1 above, most of the study respondents, 70(33.3%) had been with HIV for a period of 1 – 2 years, while most of them, 93(44.2%) had 3 to 4 biological children. Majority of them, 100(47.6%) still wanted to produce 1 to 2 more children, and this was mainly because children make one proud, 143(68.1%). All of them had ever heard about modern family planning methods, with injection, 76(36.2%) being the most known method while IUD, 37(17.6) was the most unknown modern method of family planning. Mass media (radio or television) was the commonest sources of information about modern family planning methods, 104(49.5%).

**Table 1:** Individual Characteristics of Respondents

Characteristic	Frequency (n = 210)	Percent (%)
**Age**		
Below 20 years	23	11
20–29 years	115	54.8
30–39 years	61	29
Above 40 years	11	5.2
**Sex**		
Male	34	16.2
Female	176	83.8
**Education Level**		
No formal education	29	13.8
Primary education	87	41.4
Secondary Education	90	42.9
Tertiary level	4	1.9
**Marital status**		
Single	24	11.4
Married	133	63.3
Separated/ Divorced	44	21
Widowed	9	4.3
**Religion**		
Catholic	67	31.9
Protestant	76	36.2
Muslim	47	22.3
Others	20	9.6
**Occupation**		
None	24	11.4
Self employed	128	61
Employed in public sector	58	27.6
**Duration with HIV**		
Less than a year	58	27.6
1–2 years	70	33.3
More than 2–5years	44	21
More than 5 years	38	18.1
**Number of biological children**		
1 to 2	31	14.8
3 to 4	93	44.2
More than 4	86	41
**Additional number of children wanted**		
1 to 2	100	47.6
3 to 4	96	45.7
5 or more	14	6.7
**Reason for the wanting more children**		
Other reasons	67	31.9
Many children make one proud	143	68.1
**Ever heard about Modern family planning methods**		
Yes	210	100
No	0	0
**Known modern family planning method**		
IUD	37	17.6
injection	76	36.2
Condom	38	18.1
Implant	59	28.1
**Source of information about modern family planning methods**		
Health worker	72	34.3
Radio/ TV	104	49.5
Friend	33	15.7
Other source	1	0.5

### Client-Related Factors Influencing the Utilization of Modern Family Planning Services

The only client-related factors that were found to be significantly associated with the utilization of modern FP services by PLHIV at Luwero Hospital, Luwero District were: Marital status (p = 0.032), additional number of children wanted (p = 0.037), the reasons for the wanting more children (p = 0.048), and source of information about modern family planning methods (p = 0.043), as shown in table 2.

**Table 2:** Client-related factors influencing the utilization of modern FP Services by PLHIV

Factor	Currently using FP Method						
						
	No Freq. (%)	Yes Freq. (%)	χ^2^	df	p-value	COR (CI; 95%)	AOR (CI; 95%)
**Age** (complete years)							
Below 30 years	84(40.0)	54(25.7)	1.052	1	0.305		
30 years and above	49(23.3)	23(11.0)					
**Sex**							
Male	19(9.0)	15(7.1)	0.97	1	0.325		
Female	114(54.3)	62(29.5)					
**Highest level of education**							
Below Secondary	79(37.6)	37(17.6)	2.539	1	0.111		
Secondary and above	54(25.7)	40(19.0)					
**Marital status**							
Not married	56(26.7)	21(10.0)	4.62	1	**0.032**	**1.939(1.056–3.563)** *	**2.156(1.123–4.140)** *
Married	77(36.7)	56(26.7)					
**Religion**							
Catholic	40(19.0)	27(12.9)	0.559	1	0.455		
Protestants/other religions	93(44.3)	50(23.8)					
**Occupation**							
None	14(6.7)	10(4.8)	0.292	1	0.589		
Employed	119(56.7)	67(31.9)					
**Duration with HIV**							
2 years or less	80(38.1)	48(22.9)	0.098	1	0.754		
More than 2 years	53(25.2)	29(13.8)					
**Number of biological children**							
4 or less	74(35.2)	50(23.8)	1.743	1	0.187		
More than 4	59(28.1)	27(12.9)					
**Additional number of children wanted**							
1 to 2	62(29.5)	38(18.1)	0.146	1	**0.037**	0.536(0.511–1.572) *	0.993(0.542–1.821)
3 or more	71(33.8)	39(18.6)					
**Reason for wanting more children**							
Other reasons	36(17.1)	31(14.8)	3.906	1	**0.048**	0.551(0.304–0.998) *	0.529(0.281–0.996) *
Many children make one proud	97(46.2)	46(21.9)					
**Known modern family planning method**							
Long term	60(28.6)	36(17.1)	0.053	1	0.818		
Short term	73(34.8)	41(19.5)					
**Source of information about modern family planning methods**							
Health worker	43(20.5)	29(13.8)	0.615	1	**0.043**	0.791(0.440–1.422)	
Mass media/other sources	90(42.9)	48(22.9)					

However, according to bivariate analysis, source of information about modern FP methods was not found to be significantly associated with the utilization of modern FP services (COR: 0.791, 95% CI [0.440–1.422]; p = 0.433), implying that there were confounding factors which could have led to source of information to be significantly associated with utilization of FP services by PLHIV at Luwero Hospital, Luwero District.

### Marital Status

According to bivariate analysis, marital status was found to be significantly associated with the utilization of modern FP services by PLHIV at Luwero Hospital (COR: 1.939, 95% CI [1.056–3.563], p= 0.033), implying that those who were not married were about two times more likely to use modern FP methods than those who were married. Further, on multivariate analysis, marital status was still found to be significantly associated with the utilization of modern FP services by PLHIV at Luwero Hospital (AOR: 2.2, 95% CI [1.123–4.140], p = 0.038), implying that those who were not married were at least two times more likely to use modern FP methods than those who were married.

### Additional number of children wanted

According to bivariate analysis, additional number of children wanted by the respondents was found to be significantly associated with the utilization of modern FP services by PLHIV (COR: 0.536, 95% CI [0.511–1.572], p = 0.038), implying that those who wanted 3 or more children were about two times less likely to use modern FP methods that those who wanted 1 or 2 additional children. However, on multivariate analysis, additional number of children wanted by the respondents was found not to be significantly associated with the utilization of modern FP services by PLHIV at Luwero Hospital, Luwero District (AOR: 0.99, 95% CI [0.542–1.821], p = 0.098), implying that there were confounding factors which could have led to a to be significantly associated with utilization of FP services.

### Reasons for wanting more children

According to bivariate analysis, reasons for wanting more children was found to be significantly associated with the utilization of modern FP services by PLHIV at Luwero Hospital, Luwero District (COR: 0.551, 95% CI [0.304–0.998], p = 0.04), implying that those who held the belief that many children make one proud were about two times less likely to use modern FP methods that those who didn't have that belief. Further, on multivariate analysis, reasons for wanting more children were still found to be significantly associated with the utilization of modern FP services by PLHIV (AOR: 0.5, 95% CI [0.281–0.996], p = 0.049), implying that those who held the belief that many children make one proud were half times less likely to use modern FP methods that those who didn't have that belief.

### Community Factors Influencing the Utilization of modern FP Services by PLHIV

The community factors that influence the utilization of modern FP family planning services were determined through Pearson Chi Square (χ2) analysis and there after bivariate and multivariate analysis. Table 3 below shows the summary of results.

**Table 3:** Community Factors influencing the Utilization of Modern FP Services by PLHIV

Factor	Currently using FP Method						
						
	No Freq. (%)	Yes Freq. (%)	χ2	df	p-value	COR (CI;	95%) AOR (CI; 95%)
**Culture supports the use of modern FP**							
Yes	108(51.4)	64(30.5)	0.121	1	0.728		
No	25(11.9)	13(6.2)					
**Why culture doesn't support use of FP**							
Cherishing children & for expansion of families	31(14.8)	16(7.6)	0.18	1	0.672		
N/A (supports)	102(48.6)	61(29.0)					
**Some friends using modern FP methods**							
Yes	126(60.0)	72(34.3)	0.137	1	0.711		
No or not sure	7(3.3)	5(2.4)					
**Friends discourage the use of modern FP methods**							
Yes	73(34.8)	47(22.4)	0.754	1	0.385		
No	60(28.6)	30(14.3)					
What friends say about FP methods							
They say negatives	73(34.8)	47(22.4)	0.754	1	0.385		
N/A	60(28.6)	30(14.3)					
**Community talks bad about the people who use modern FP methods**							
Yes	82(39.0)	48(22.9)	0.922	1	0.01	0.971(0.545–1.732)	
No/Not sure	51(24.3)	29(13.8)					
**Religion discourages the use of modern FP methods**							
Yes	102(48.6)	63(30.0)	0.761	1	**0.038**	**0.631(0.361 -1.480)** *	1.981(0.622–0.816)
No/Not sure	31(14.8)	14(6.7)					
**Religious views on modern FP methods**							
It's against Gods will & should multiply to fill the earth	102(48.6)	64(30.5)	1.216	1	**0.027**	**0.618(0.571–1.171)** *	**0.661(0.282–0.616)** *
N/A	31(14.8)	13(6.2)					
**Presence of organizations or people that provide FP services**							
Yes	132(62.9)	76(36.2)	0.155	1	**0.694**		
No	1(0.5)	1(0.5)					
**Organizations or people present in the area**							
Private clinics/NGOs	132(62.9)	76(36.2)	0.155	1	0.694		
N/A (No Organisations)	1(0.5)	1(0.5)					

* p < 0.05

According to the results in table 3 above, basing on Pearson Chi Square (χ2) analysis, the only community factors that were found to be significantly associated with the utilization of modern FP services by PLHIV at Luwero Hospital were: community talking negatively about people who use modern FP methods (p = 0.01), religion discouraging the use of modern FP methods (p = 0.038) and religious views on modern FP methods (p = 0.027). However, according to bivariate analysis, community talking negatively about people who use modern FP methods was not found to be significantly associated with the utilization of modern FP services at Luwero Hospital (COR: 0.971, 95% CI [0.545–1.732], p = 0.922), implying that there were confounding factors which could have led to negative talk from community to be significantly associated with utilization of FP services PLHIV.

### Religion Discouraging the Use of Modern FP Methods

According to bivariate analysis, religion discouraging the use of modern FP methods was found to be significantly associated with the utilization of modern FP services by PLHIV at Luwero Hospital, Luwero District (COR: 0.631, 95% CI [0.361–1.480], p = 0.038), implying that those who held the belief that religion discourages the use of modern FP methods were about two times less likely to use modern FP methods that those who didn't have that belief. However, on multivariate analysis, religion discouraging the use of modern FP methods was found not to be significantly associated with the utilization of modern FP services (p = 0.101).

### Religious Views about Modern FP Methods

According to bivariate analysis, religious views on modern FP methods was found to be significantly associated with the utilization of modern FP services by PLHIV at Luwero Hospital, Luwero District (COR: 0.618, 95% CI [0.571–1.171], p = 0.034), implying that those who held the belief that FP is against God's will and that their religion commands them to multiply to fill the earth were about half times less likely to use modern FP methods that those who didn't have that belief. Further, on subjecting to multivariate analysis, religious views on modern FP methods were found to be significantly associated with the utilization of modern FP services by PLHIV at Luwero Hospital (AOR: 0.7, 95% CI [0.282–1.616], p = 0.034), implying that those who held the belief that FP is against God's will and that their religion commands them to multiply to fill the earth were about half times less likely to use modern FP methods than those who didn't have that belief.

### Method-Related Factors Influencing the Utilization of modern FP Services by PLHIV

The method-related factors that influence the utilization of modern FP family planning services were determined through Pearson Chi Square (χ2) analysis and there after bivariate and multivariate analysis. Table 4 below shows the summary of results.

**Table 4:** Method-Related Factors Influencing the Utilization of Modern FP Services by PLHIV

Variables	Currently using FP Method						
					
	No Freq. (%)	Yes Freq. (%)	χ2	Df	p-value	COR (CI; 95%)	AOR (CI; 95%)
**Has used modern family planning method before**							
Yes	131(62.4)	74(35.2)	1.201	1	0.273		
No	2(1.0)	3(1.4)					
**Modern family planning method used before**							
Long term	62(29.5)	30(14.3)	1.161	1	0.281		
Short term or N/A (Has never used)	71(33.8)	47(22.4)					
**Challenges related to the side effects of using modern FP methods**							
Yes	125(59.5)	67(31.9)	3.025	1	**0.022**	**2.332(0.879–6.188)** *	**1.756(0.043–0.168)** *
No/Not applicable	8(3.8)	10(4.8)					
**Found it easy to use modern family planning methods**							
Yes	108(51.4)	63(30.0)	0.912	1	**0.012**	0.960(0.465–1.981)	
No/Not applicable	25(11.9)	14(6.7)					
**Method, one has found easy**							
Long term	57(27.1)	34(16.2)	0.033	1	0.855		
Short term or N/A	76(36.2)	43(20.5)					

* p < 0.05

** p < 0.01

### Challenges Related to Side Effects of Using Modern Family Planning Methods

According to the results in table 4 above, basing on Pearson Chi Square (χ2) analysis, the only method-related factors that were found to be significantly associated with the utilization of modern FP services by PLHIV at Luwero Hospital were: challenges related to the side effects of using family planning methods (COR: 2.33, 95% CI [0.879–6.188], p = 0.022) and finding it easy to use modern family planning methods (p = 0.012). Further, on subjecting to multivariate analysis, challenges related to side effects of using FP methods were still found to be significantly associated with the utilization of modern FP services by PLHIV at Luwero Hospital, Luwero District (AOR: 1.8, 95% CI [0.043–1.968], p = 0.044), implying that those who reported challenges related to side effects of using FP were about two times more likely to use modern FP methods than those who didn't report such challenges.

### Service Provision-Related Factors Influencing the Utilization of modern FP Services by PLHIV

The service provision-related factors that influence the utilization of modern FP family planning services were determined through Pearson Chi Square (χ2) analysis and there after bivariate and multivariate analysis. Table 5 below shows the summary of results.

**Table 5:** Service Provision-Related Factors Influencing the Utilization of Modern FP Services by PLHIV

Factor	Currently using FP Method						
						
	No	Yes	χ^2^	df	p-value	COR (CI; 95%)	AOR (CI; 95%)
	Freq. (%)	Freq. (%)					
**Awareness that the hospital provides family planning services to persons living with HIV**							
Yes	130(61.9)	75(35.7)	0.876	1	**0.025**	**0.601(0.189–7.072)** *	1.144(0.061–0.817)
No	3(1.4)	2(1.0)					
**Services friendly for persons living with HIV**							
No/Not aware	15(7.1)	20(9.5)	7.583	1	**0.006**	**2.760(1.316–5.787)** **	**2.406(0.022–0.582)** *
Yes	118(56.2)	57(27.1)					
**Reasons for services not being friendly**							
Rude health workers/Long waiting hours	12(5.7)	23(11.0)	8.298	1	**0.016**	**1.560(0.373 -0.843)** *	0.577(0.110–0.798)
N/A (services friendly or not known)	118(56.2)	57(27.1)					
**Received counselling or advise about family planning method**							
Yes	129(61.4)	75(35.7)	0.03	1	0.864		
No	4(1.9)	2(1.0)					
**Availability of family planning methods at the hospital when needed**							
Yes	130(61.9)	75(35.7)	0.025	1	0.876		
No/Not sure	3(1.4)	2(1.0)					
**Cost related challenges in regard to the use of modern family planning methods**							
Yes	82(39.0)	53(25.2)	1.094	1	0.296		
No/Not applicable	51(24.3)	24(11.4)					
**The costs involved**							
High cost of transport and nutrition demands	82(39.0)	53(25.2)	1.094	1	0.296		
N/A	51(24.3)	24(11.4)					
**Missed family planning method due to supplies missing at the hospital**							
Yes	44(21.0)	26(12.4)	0.01	1	0.919		
No	89(42.4)	51(24.3)					

* p < 0.05

** p < 0.01

According to the results in table 5 below, basing on Pearson Chi Square (χ2) analysis, the only service provision-related factors that were found to be significantly associated with the utilization of modern FP services by PLHIV at Luwero Hospital: awareness that the hospital provides FP services to PLHIV (p = 0.025), services being friendly for PLHIV (p = 0.006), and reasons for services not being friendly (p = 0.016).

### Awareness That the Hospital Provides FP Services to PLHIV

According to bivariate analysis, awareness that the hospital provides FP services to PLHIV was found to be significantly associated with the utilization of modern FP services by PLHIV (COR: 0.601, 95% CI [0.189–7.072], p = 0.035), implying that those who were aware were about half times less likely to use modern FP methods that those who were not aware of the existence of these services. However, on subjecting to multivariate analysis, awareness that the hospital provides FP services to PLHIV was found not to be significantly associated with the utilization of modern FP services by PLHIV (AOR: 1.1, 95% CI [0.061–1.817], p = 0.546), implying that there were confounding factors which could have led to awareness that the hospital provides FP services to PLHIV to be significantly associated with utilization of FP services by PLHIV at Luwero Hospital, Luwero District.

### Services Being Friendly for PLHIV

According to bivariate analysis, FP services being friendly for PLHIV was found to be significantly associated with the utilization of modern FP services by PLHIV (COR: 2.760, 95% CI [1.316–5.787], p = 0.007), implying that those where FP services were friendly for PLHIV were at least 1.3 times more likely to use modern FP methods than those for whom services were not friendly. Further, on subjecting to multivariate analysis, FP services being friendly for PLHIV was still found to be significantly associated with the utilization of modern FP services by PLHIV (AOR: 2.4, 95% CI [0.022–2.582], p = 0.00).

### Reasons for Services Not Being Friendly

According to bivariate analysis, the reasons for FP services not being friendly to PLHIV was found to be significantly associated with the utilization of modern FP services by PLHIV at Luwero Hospital (COR: 1.560, 95% CI [0.373–1.843], p = 0.025), implying that those who experienced rude health workers and/or long waiting hours were about 1.5 times more likely to use modern FP methods, despite the negative experiences.

### Summary of Results

The study found that despite the presence of modern family planning services at Luwero Hospital, the uptake of these services is low (at 36.7%) among persons living with HIV. Basing on Pearson Chi Square (χ2) analysis, the client-related factors that are significantly associated with the utilization of modern FP services by PLHIV at this hospital were: Marital status (p = 0.032), additional number of children wanted (p = 0.037), the reasons for the wanting more children (p = 0.048), and source of information about modern family planning methods (p = 0.043). The only community factors found to be significantly associated with the utilization of modern FP services by PLHIV at this hospital were: community talking negatively about people who use modern FP methods (p = 0.01), religion discouraging the use of modern FP methods (p = 0.038), and religious views on modern FP methods (p = 0.027).

The only method-related factors were: challenges related to the side effects of using family planning methods (p = 0.022) and finding it easy to use modern family planning methods (p = 0.012). The only service provision-related factors were: awareness that the hospital provides FP services to PLHIV (p = 0.025), services being friendly for PLHIV (p = 0.006), and reasons for services not being friendly (p = 0.016).

Further, on multivariate analysis, marital status was still found to be significantly associated with the utilization of modern FP services by PLHIV at Luwero Hospital (AOR: 2.2, 95% CI [1.123–4.140], p = 0.038), implying that those who were not married were at least two times more likely to use modern FP methods than those who were married. FP services being friendly for PLHIV was still found to be significantly associated with the utilization of modern FP services by PLHIV (AOR: 2.4, 95% CI [0.022–2.582], p = 0.00).

## Discussions

### Client-Related Factors Influencing the Utilization of Modern FP Services by PLHIV

The study found that marital status significantly influenced the utilization of modern FP services by PLHIV at Luwero Hospital. Those who were not married were at least two times more likely to use modern FP methods than those who were married (AOR: 2.2; p = 0.038). Probably those who were not married considered themselves more at risk of unintended pregnancy than their counterparts who were married. Those who were married could have been more likely to use modern FP methods because marital obligations predisposes them to the risk of unintended pregnancies, hence the desire to use modern FP methods to protect themselves accordingly. This finding contradicts that of Magadi and Magadi^18^ who in a study conducted in Kenya found that the use of modern FP method was more common among married PLHIV than their unmarried counterparts. The finding is similar to that of Warren et al^19^ in a study about family planning practices and pregnancy intentions among HIV-positive and HIV-negative postpartum women in Swaziland. It was found that PLHIV who were married had higher chances of using modern family planning services than those who were not married.

For this current study, additional number of children wanted by the respondents was found to be significantly associated with the utilization of modern FP services by PLHIV at Luwero Hospital. Those who had the desire to produce 3 or more children were about half times less likely to use modern FP methods than those who wanted less number of children (COR: 0.536; p = 0.038); probably, the desire for additional number of children forced them not to desire to use modern FP method. This finding is supported by another study in Kenya^20^ which also reported lower rates of utilization of modern family planning methods among PLHIV who had the desire to produce more children that those who didn't have such desire. The finding is also supported by Egessa^21^ who in conceptual model on family planning and HIV to sexually active PLHIV in Uganda reported higher fertility intensions as a hindrance to uptake of modern FP services. In the current study, on multivariate analysis however, additional number of children wanted by the respondents was found not to be significantly associated with the utilization of modern FP services by PLHIV, which is in line with the study by Pokharel et al 22 in Kathmandu, Nepal, in which fertility intentions were not found to significantly influence utilization of modern FP services by PLHIV. Again, in this current study, those who held the belief that many children make one proud were about half times less likely to use modern FP methods that those who didn't have that belief (p = 0.049). Probably this is attributed to the inherent desire for children^23^. The desire for children as a source of pride could have negatively influenced PLHIV on use of modern FP methods.

### Community Factors Influencing the Utilization of Modern FP Services by PLHIV

According to bivariate analysis, those who held the belief that religion discourages the use of modern FP methods were about half times less likely to use modern FP methods than those who didn't have that belief (COR: 0.631, p = 0.038). Probably, this is attributable to the fact that some religions publicly discourage the use of modern family planning methods^24^. This finding is supported by Ukwuani et al 25 who also reported that religious beliefs in some communities are a hindrance to the use of modern family planning utilization. This could have been the reason as to why in the current study, religious views on modern FP methods was found to be significantly associated with the utilization of modern FP services by PLHIV at Luwero Hospital, Luwero District (p = 0.034), especially the belief that modern FP is against God's will and that their religion commands them to multiply to fill the earth. However, on multivariate analysis, religion discouraging the use of modern FP methods was found not to be significantly associated with the utilization of modern FP services (AOR: 1.981; p = 0.101), implying that there were confounding factors which could have led to additional number of children being significantly associated with utilization of FP services by PLHIV. This finding is also similar to other community factors elsewhere, especially among the Ik community of Kaabong district, Uganda 26.

### Method-Related Factors Influencing the Utilization of Modern FP Services by PLHIV

The study found that challenges related to side effects significantly influenced the utilization of modern FP services by PLHIV at Luwero Hospital, Luwero District. Those who reported experiencing challenges related to side effects of using FP were about two times less likely to use modern FP methods that those who didn't report such challenges (AOR: 1.756; p = 0.044). Probably such side effects are not easy to bear, thereby acting as hindrances to utilization of modern FP methods. This finding is however, supported by Savabi-Esfahany et al^27^ and Barden-O'Fallon et al 28 who also reported that method-related problems were some of the most common reasons for discontinuation of use of modern family planning methods.

### Service Provision-Related Factors Influencing the Utilization of Modern FP Services by PLHIV

The current study found that awareness about availability of FP services significantly influenced the utilization of modern FP services by PLHIV at Luwero Hospital. Those who were aware about the availability of such services were about half times less likely to use modern FP methods than those who were not aware of the existence of these services (COR: 0.601; p = 0.035). This is probably an indication that health workers were sensitizing them about the existence of the services which could have empowered them to try the services. This finding is supported by Simba et al 29 who also acknowledged sensitization by health workers as playing an active role in supporting the uptake of modern FP services among PLHIV. On multivariate analysis, however, awareness about availability of FP services was did not significantly influence the utilization of modern FP services by PLHIV at Luwero Hospital (AOR: 1.144; p = 0.546). Friendliness of services to PLHIV was found to significantly influence the utilization of modern FP services by PLHIV at Luwero Hospital. Those who reported FP services as being friendly were at least two times more likely to use modern FP methods than those for whom the services were not friendly (AOR: 2.406; p = 0.00). Probably, the services being friendly is empowering and motivating enough for PLHIV to consider utilizing them. This finding is however, supported by Ika, Okello & Omona^30^ who studied elimination of mother to child transmission in Arua and also supported by Hutchison et al 31 who reported service friendliness as being key to improving the uptake of health services.

In the current study, some respondents reported experiencing rude health workers and/or long waiting hours, which negatively influenced their utilization of modern FP services (p = 0.025). This finding is in line with Hutchison et al31 who reported poor uptake of modern FP services among PLHIV due to rude health workers.

## Conclusion

The study found that despite the presence of modern family planning services at Luwero Hospital, the uptake of these services is low among persons living with HIV. The low uptake of FP attributed to client-related factors, community factors (such as religious views discouraging the use of modern family planning), method-related factors (such as negative side effects) and service provision-related factors (such as services being unfriendly for persons living with HIV) need to be improved. Poor utilization of modern family planning methods by persons living with HIV is a recipe for unintended pregnancy and thus jeopardizes efforts in elimination of mother-to-child transmission of HIV, especially the second prong which emphasizes the prevention of unwanted pregnancies among women living with HIV.

## Recommendations

The Authors therefore recommend Government of Uganda, through the ministry of health to engage with religious leaders regarding the utilization of modern family planning methods. The health facility should engage with and create partnerships with male partners of women living with HIV to ensure that such men are supportive of their partners' utilization of modern family planning services. The health facility leadership and management need to follow-up and supervise their health staff to ensure improved modern family planning service provision environment for persons living with HIV. Lastly, health workers should ensure that adequate information are given to clients regarding the side effects of modern family planning methods especially on the management of such challenges.

## Authors' Information

OK is a medical doctor, lecturer and researcher in the Faculty of Health Sciences (FHS) of Uganda Martyrs University. He holds a PhD in Management (Healthcare Mgt), MSc. HSM, MSc. M & E, PGD PPM and MBChB. MG is a lecturer and MPH Specialist working with Kampala University, Department of Public Health, Kampala, Uganda.
